# Experiences with a researcher-centric ELN†
†Electronic supplementary information (ESI) available. See DOI: 10.1039/c4sc02128b



**DOI:** 10.1039/c4sc02128b

**Published:** 2014-10-20

**Authors:** Katrina A. Badiola, Colin Bird, William S. Brocklesby, John Casson, Richard T. Chapman, Simon J. Coles, James R. Cronshaw, Adam Fisher, Jeremy G. Frey, Danmar Gloria, Martin C. Grossel, D. Brynn Hibbert, Nicola Knight, Lucy K. Mapp, Luke Marazzi, Brian Matthews, Andy Milsted, Russell S. Minns, Karl T. Mueller, Kelly Murphy, Tim Parkinson, Rosanne Quinnell, John S. Robinson, Murray N. Robertson, Michael Robins, Emma Springate, Graham Tizzard, Matthew H. Todd, Alice E. Williamson, Cerys Willoughby, Erica Yang, Paul M. Ylioja

**Affiliations:** a School of Chemistry , The University of Sydney , NSW 2006 , Australia; b Chemistry , University of Southampton , Southampton , SO17 1BJ , UK . Email: J.G.Frey@soton.ac.uk; c OptoElectronics Research Centre , University of Southampton , SO17 1BJ , UK; d Scientific Computing Department , STFC Rutherford Appleton Laboratory , Chilton , Didcot , Oxfordshire OX11 0QX , UK; e Central Laser Facility , STFC Rutherford Appleton Laboratory , Chilton , Didcot , Oxfordshire OX11 0QX , UK; f School of Chemistry , UNSW Australia , Sydney , NSW 2052 , Australia; g Penn State University , Department of Chemistry , University Park , 104 Chemistry Building , PA , USA; h Electronics and Computer Science , University of Southampton , Southampton , SO17 1BJ , UK

## Abstract

Electronic Laboratory Notebooks (ELNs) are progressively replacing traditional paper books in both commercial research establishments and academic institutions. University researchers require specific features from ELNs, given the need to promote cross-institutional collaborative working, to enable the sharing of procedures and results, and to facilitate publication. The LabTrove ELN, which we use as our exemplar, was designed to be researcher-centric (*i.e.*, not only aimed at the individual researcher's basic needs rather than to a specific institutional or subject or disciplinary agenda, but also able to be tailored because it is open source). LabTrove is being used in a heterogeneous set of academic laboratories, for a range of purposes, including analytical chemistry, X-ray studies, drug discovery and a biomaterials project. Researchers use the ELN for recording experiments, preserving data collected, and for project coordination. This perspective article describes the experiences of those researchers from several viewpoints, demonstrating how a web-based open source electronic notebook can meet the diverse needs of academic researchers.

## Abstract 2

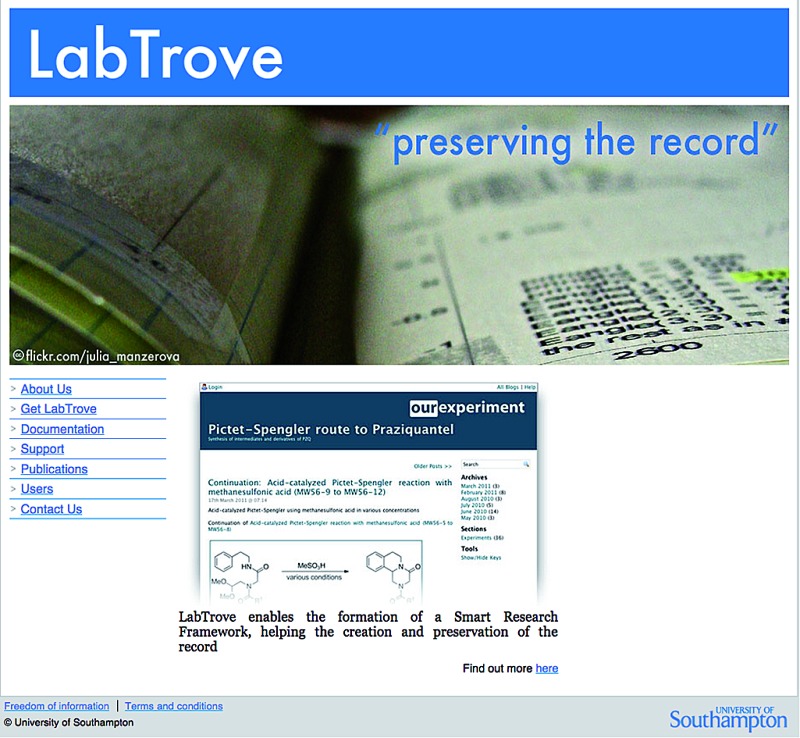
Chemical scientists’ experiences with LabTrove.

## 1 Introduction

Electronic Laboratory Notebooks (ELNs) are progressively replacing traditional paper books for keeping the experimental record,^1^ especially in commercial research establishments. Important drivers for this change are the need to comply with regulatory requirements and the desire to protect and expand intellectual property (IP); structured notebook systems should provide the necessary audit trails.^2^ ELNs offer considerably more to researchers and to research groups than do systems that offer only content management and sharing facilities. ELNs unite the objects that comprise the record of research and preserve the provenance of each object and its contribution to the overall narrative.

The 2013 review of laboratory notebooks in the digital era (by three of the authors)^1^ not only explores the history and the expanding use of electronic laboratory notebooks but also presents the results of a comprehensive survey of the literature relating to ELNs. It is clear from the survey and from considerations of the scientific applications for which ELNs have been deployed that digital notebooks come in a range of embodiments. The distinction between commercial and academic institutions is but one aspect; in many cases the designers of ELNs have adapted both the input and output to match the specific applications for which the ELNs are used. Most ELNs, especially the major commercial offerings, are proprietary. There is, however, increasing interest in open source implementations, as they enable individual laboratories or institutions to tailor the operation and the data preservation to suit their particular needs. The review also examines the social and technical issues that combine to influence moves from paper to digital notebooks.

There are many concerns in the academic environment with the adoption of digital notebooks. The obvious issues of cost are significant but more fundamental are the concerns with the appropriateness of the function. A view frequently expressed is that the systems have been designed and have evolved for commercial environments, and for large companies with uniform practices. The question also arises whether the concept of a digital notebook fits with the diversity of the academic research environment. Within laboratory chemistry, there is at least the concept of a notebook. In many computationally based projects, even the notebook is not rigorously used.

While sharing the need for audit trails and secure data handling, academic laboratories require a more flexible means of recording experiments and one that is more researcher-centric (*i.e.*, not only aimed at the individual researcher's basic and fundamental needs rather than tailored to a specific institutional or subject/disciplinary agenda, but also able to be tailored because it is open source) and appropriate to the obligation of research institutions to champion original research. In science, much of this work is *via* cross-institutional collaboration and so any ELN solution has to enable the sharing of procedures and results, and must facilitate publication. Scientific progress depends on adaptability and sharing, leading to a preference within certain academic environments for open source tools: ELNs should be no exception. Moreover, the cost of an ELN can be a significant issue for academic researchers. For some, cost considerations favour a non-proprietary solution that can be tailored to particular needs in preference to a comprehensive but expensive system that might be closed or difficult to tailor. This is particularly pertinent for departments that have regulated paper notebook systems, as there may be resistance from administrators to any additional expenditure. However, as Brynn Hibbert notes, the cost of providing formal laboratory notebooks can be significant.^3^

Traditional laboratory notebooks are still in widespread use for documenting experiments. Notebook entries include plans and designs, recording the experimental method and any changes to the experimental design. Entries are dated and in chronological order. Data are kept as printouts from instruments and are stapled or glued into the notebook. As such, paper records are not ideal for sharing but do capture the provenance of the research. As it becomes more common for instruments to be connected to networks it will be essential to work with the digital files directly rather than produce printouts to stick into paper notebooks. Data from instruments can already be collected by some ELN systems, using for example an application programming interface (API) to enter data programmatically into the ELN.

Universities must also be aware of the need to train their students in the technologies and systems that they will encounter when they become employed and to improve their own record keeping systems. The preferred approach is to ensure students understand the importance of thorough record keeping, whatever the demands of their individual studies, and encouraging them to learn with a flexible notebook system that can be easily adapted for different student needs in preparation for industrial and commercial environments where procedures are of necessity more uniform.

**Fig. 1:**
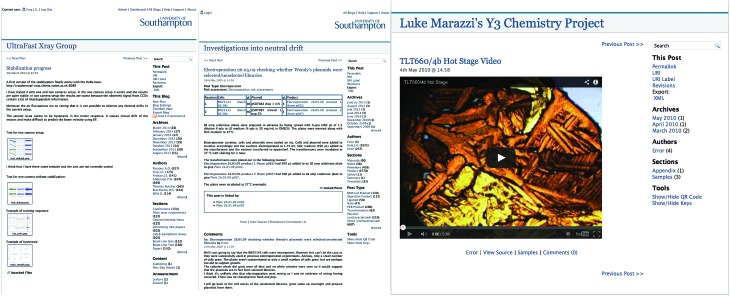
Typical LabTrove notebook entries, illustrating how researchers have supplemented their journal recording with embedded graphics, tables, and inline imagery: The Ultrafast Xray Group's laser based soft X-ray imaging example is taken from a closed group project Trove that has been in use for over 5 years;^5^ The chemical biology investigation of neutral drift is taken from one of the early open notebook science projects directed by Cameron Neylon;^6^ The Y3 chemistry example is taken from Luke Marazzi's undergraduate project in crystallography at the University of Southampton and is publicly visible.^7^

LabTrove was designed and developed as an open source, web-based recording system that can be tailored to the needs of the individual researcher, while maintaining appropriate levels of security, and capturing the *meta*-information necessary to establish reliable provenance.^4^ LabTrove is blog-based, thus preserving the chronological journal characteristic of traditional notebooks, while properly indexing and cross referencing the relevant entries in the repository and making them freely viewable and citeable in scholarly publications (Fig. 1).

### 1.1. Terminology

Appendix A provides a list of all abbreviations used in this paper. We use the following terminology for the components of a LabTrove installation (Table 1).

**Table 1:** Terminology for LabTrove components

Trove	A single LabTrove installation comprising any number of notebooks that can optionally be grouped according to their usage
Notebook	A collection comprising any number of *entries* and a collection of metadata keys
Entry	The LabTrove representation of a blog post, which can have any number of attached data files and any number of associated comments

### 1.2. LabTrove

LabTrove is open source, meaning that its users can adapt its functionality to meet the disparate needs of their scientific communities. Examples are the ModTrove software from one of us (MR),^8^ and the work done by CSIRO on behalf of UNSW, which is now folded into the main branch of the ELN. The CSIRO project is notable for the routine mining of all notebook entries for chemical keywords.^3^

A recent review of the role of laboratory notebooks in the digital era demonstrates the broad scope of modern scientific record keeping and also shows the increasing importance of digital record keeping to open science and open source projects.^1^ New users can download LabTrove from the website.^9^ We have also described the use of this ELN for open notebook science^10,11^ at an American Chemical Society meeting.^12^

In this article, we compare and contrast the experiences of a diverse selection of academic laboratories operating in a broad range of environments. We discuss attitudes to the use of ELNs in general and to LabTrove in particular, and evaluate the use of this ELN for specific application areas, including teaching and learning. We also appraise the reticence shown towards capturing metadata, although we examine the subject of metadata in ELNs at greater depth in a separate article.^13^

Robertson *et al.* ^14^ report the use of LabTrove in an open science drug discovery project, Open Source Malaria (OSM), as described elsewhere in this article. They also comment on the merits of other tools, such as wikis.^15^ While the ELN provides the means to share all primary data, which is fundamental to open projects, a wiki can have advantages for project coordination, for example in providing a single status page that is maintained continually, and when preparing publications.

We begin by reviewing how LabTrove has been used: the range of scientific disciplines; the ways in which groups deploy the ELN; how they use it to facilitate communication and collaboration; and how groups organise their projects and the notebooks within them. We discuss the attitudes of researchers to the use of ELNs in general, we appraise the devices used in conjunction with researcher input; and then examine a range of specific application areas.

Two highlighted features of the exemplar ELN are its flexible metadata support and its provision of templates to assist data entry for repeated or parallel procedures.^4^ We evaluate the use of these two features by researchers in the various groups. In the concluding sections, taking the view that in this article we are effectively reporting a number of case studies, we consider the implications for ELNs in general.

This perspective article describes the experiences of those researchers from several points of view, demonstrating how an electronic notebook can meet the diverse needs of academic research groups and individual researchers. Detailed consideration of individual projects is outside the scope of this article, but is supplied by the associated references. In this article we discuss the impact that adoption of a digital notebook has had on several groups within physical science research. The focus of the article is to illustrate advantages, and of course issues, with what is a major shift in the way research students and staff record their research.

## 2 Usage synopsis: how the ELN has been used

LabTrove was designed to be a flexible system for recording scientific research in an electronic laboratory notebook, retaining the journal characteristics of traditional notebooks while exploiting the potential for linking together procedures, materials, samples, observations, data, and analysis reports.^4^

The experiences of individual researchers and research groups that we report derive from using the ELN for a wide range of scientific purposes, several being interdisciplinary. These scientific disciplines comprise: analytical chemistry, medicinal and synthetic organic chemistry, physics, laser-based studies, biological evaluations, computational chemistry, process engineering, undergraduate and postgraduate teaching.

Usage descriptions provided directly by users of the ELN illustrate its flexibility and the importance of its researcher-centric approach. For example, analytical chemists capture large amounts of routine measurements from a variety of instruments; a biomaterials project uses the ELN to organise separate experiments collaboratively; a multidisciplinary project coordinates research in several laboratories; for another project, LabTrove is the source of a data processing pipeline. The usage overall reveals a range of requirements and choices. By design LabTrove is not prescriptive about the content of any type of entry, whether it recounts an experiment or some other aspect of a researcher's workflow. Entries that describe experiments usually reflect the nature of the research, for example observers of long-running biological experiments create entries manifestly different from those produced by synthetic organic chemists.

### 2.1. How groups have deployed the LabTrove ELN

The researchers report a range of requirements and choices regarding their manner of using the LabTrove ELN.

At the University of Sydney, Mat Todd's group has used LabTrove for both their current open notebook science projects; only closed projects employ traditional paper-based lab notebooks (and some use private Troves as well). Todd is a notable pioneer of open science, having coordinated his group's research into a new asymmetric route to the drug Praziquantel in public view using LabTrove.^16–18^ This ELN also forms the central mechanism for data sharing in the Open Source Malaria project that involves researchers worldwide.^19^

Members of the Todd group who are involved in open projects, and researchers based in other laboratories, are expected to record raw data and procedures immediately; the later writing up of notes taken on pieces of paper is strongly discouraged. Subsequent editing of entries is straightforward and acceptable when combined with a comprehensive revision history. The two open source projects coordinated by the group have involved approximately thirty people each, participating from various locations *via* the ELN or other online resources. In other laboratories, the most common practice is to limit access to group members, with provisions for contributions from guest collaborators (who may be external to the institution).

The biomaterials project involves synthesising polymers for incorporation into biologically relevant materials, so ranges in scope from organic synthesis to surface modification and rheological measurements. This project uses LabTrove as an alternative to using a paper notebook, especially as the ELN facilitates collaboration with colleagues conducting cell-based studies in the local hospital. Demonstrating reproducibility between batches of sample was an important aspect of this collaboration, which relied on the linking and classification features of the ELN. Analyses of coated surfaces could be linked to the separate batches and also to the master entry for all the batches, enabling the project to keep track of separate samples and to see the overall picture in terms of surface comparison and reproducibility.

LabTrove is a key resource for the UK National Crystallography Service, as it complements the Laboratory Information Management System (LIMS) that the service uses to manage samples and the data repository in which it stores the results of experiments. Crystallographic work is naturally very collaborative: characterising samples synthesised by other researchers and incorporating the results back into the broader research programme. The complementary deployment of LabTrove enables the service to meet the challenges associated with: the provenance of samples and data; obtaining the information provided by other techniques; discussing results and their interpretation; and creating cohesive publications for which sections are written by individuals in isolation.

Some institutions have existing policies with regard to laboratory notebooks. For example, The University of New South Wales (UNSW) requires all research degree students to use a hard-backed laboratory notebook with numbered pages: these books remain the property of the School of Chemistry. By special arrangement, researchers in Brynn Hibbert's group have the option to use the ELN in conjunction with the hard-copy book, or instead of it. Some students make extensive use of LabTrove; one student used it exclusively for his PhD, with no paper record at all.

Students who have used both the ELN and paper notebooks comment on the inefficiency of the paper notebook, especially with regard to curating data that were created in a digital form. The real risks of damage to paper records are also a concern. Searching for records and sharing results with colleagues are both activities that are easier and more efficient and reliable with an ELN than with paper-based records of experiments. One researcher who had in the past had to go back to paper after using an ELN described the experience as “painstaking” and the record keeping as “inefficient by comparison.”

All researchers involved in the Ultrafast X-ray studies^6^ contribute to the project Trove: staff, fellows, postgraduates, project students, and summer interns. All are strongly encouraged to add material daily and certainly as a summary prior to each weekly group meeting, as the Trove is used as a project resource, providing a record and collaboration system for the project rather than for the notebooks of individual researchers.

The Mueller group at The Pennsylvania State University (Penn State)^20^ derives benefits from both group and individual notebooks. The group notebook provides a database for material discussed at group meetings and, importantly, preserves access to information originated by group members who have since moved on. Individual notebooks provide information for dissemination to collaborators and, because they are online, avoid the need to carry paper notebooks when travelling.

Our separate study of metadata usage in LabTrove took account of the underlying purpose of each notebook.^13^ The majority of the notebooks that we surveyed are used for recording experiments, with other notebooks being used for experiment-related data such as materials catalogues and instrument descriptions. Some groups use the ELN for administrative purposes, such as recording project and personal activities, testing of and discussion about LabTrove itself; a small number of notebooks are used for multiple purposes, comprising both experimental data and project information.

The numbers of notebooks created and used varies according to local practices, so notebooks might be essentially personal or be shared by the researchers working on a group project. Approximately 10% of the notebooks established at UNSW are currently active, owing to projects having been completed. Groups preserve the records of researchers who have graduated or moved on for other career reasons. The Todd group takes a snapshot of their ELN at specific points in time, such as when a paper is published. The snapshot is stored in an institutional repository and can be downloaded subsequently.^21^

LabTrove is also used at the Science & Technology Facilities Council (STFC) Central Laser Facility (CLF) at the Rutherford Appleton Laboratory (RAL) by staff and users of the Artemis facility. The ISIS group at STFC uses the ELN to enable scientists to keep track of experiment status and the data reduction workflow for a live experiment. The data provenance includes sample information and links between samples, experiment data, derived data, and subsequent publications.

### 2.2. How an ELN facilitates communication and collaboration

For all groups, the ELN provides a central repository for data and other information, which can be accessed outside the laboratory *e.g.* during progress meetings and when updating the project from other locations. For the Todd group, the key advantage of electronic storage is that all the data associated with an experiment are kept in one place, with the experiment. The X-ray group runs its meetings from the date-ordered view of their Trove, by going through the entries added since the previous meeting. Communication in the nanodroplets group, when working at the Central Laser Facility in collaboration with researchers across different sites, is strongly dependent on the ELN, which:

…serves as an important way to communicate up to the minute news on the progress of the experiment to people not on site and, as a way to keep in contact about the data analysis once the experiment time is over.

The Trove provides a place for discussion of plans, a repository for some data and the discussion of that data, and a place to ask for opinion or advice. The Trove therefore allows people who are remote to each other, and the actual experiment, access to up to the minute information on the progress and, often more importantly, any problems encountered. They can then feed back thoughts, ideas and comments with the Trove then becoming an open forum for discussion.

The Todd group captures communications between individual team members that may have occurred outside the ELN, by for example uploading the text of e-mails that contain data or discussions about data. Their ELN also stores shared files such as “How To” guides and meeting minutes. Longer project discussions and other project outputs are usually stored elsewhere and linked from the associated entry, but the Open Source Malaria (OSM) group does maintain a project notebook on their Trove.

### 2.3. How groups organise their projects and notebooks

LabTrove was designed to be flexible with regard to the needs of individual researchers. Users organise their Troves and notebooks in a variety of ways, ranging from the journal style reminiscent of the traditional paper notebook to a more formal and structured approach, in some instances involving templates.

The approach adopted by the Todd group is broadly representative with respect to organisation, although less so regarding content. A typical entry from a member of the synthetic team includes:

• a title, with a brief description of the purpose of the experiment, thus using the ELN as a journal;

• a reaction scheme, prepared using ChemDraw^22^ and embedded as an image file;

• a table containing names, quantities, and other important values for the chemicals used, constructed in Excel and added as a data file in PNG or other suitable format;

• the risk assessment procedure as a PDF file.

The complete model entry is available from: http://malaria.ourexperiment.org/osm_logos_and_templ/7788/post.html.

**Fig. 2:**
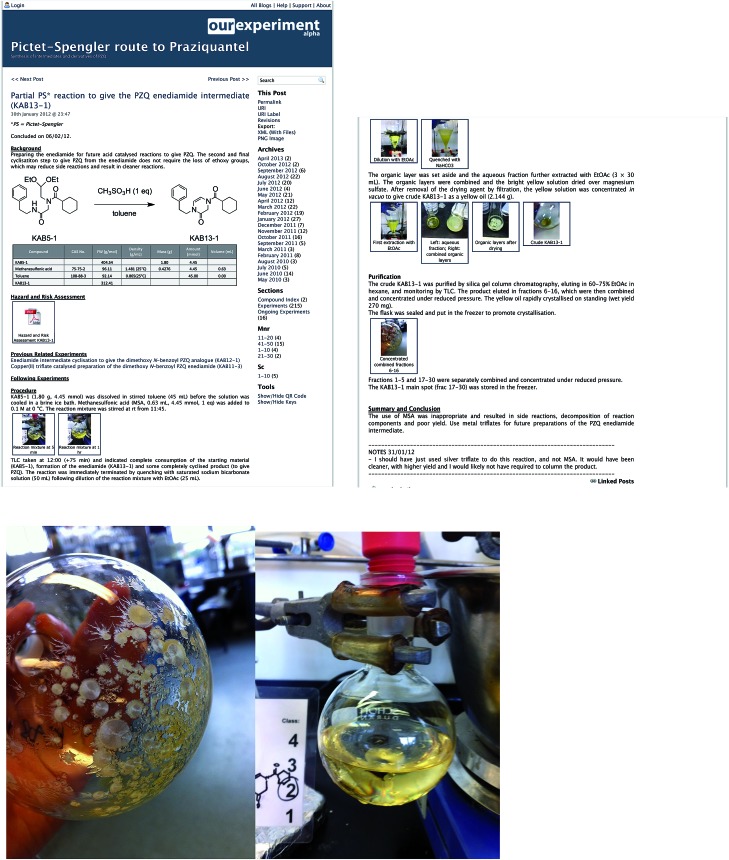
Examples of the use of photos in a laboratory notebook entry from the Open Source Malaria (OSM) project,^19^ shown as thumbnails in the main entry, but which can be expanded to full size and downloaded and are also listed at the bottom of the entry with all attached files, not all of which have to be chosen to be made visible in the body of the entry.^23^

Mat Todd regards photographs of experiments as a major part of the advantage of the ELN over paper notebooks, for example photos of thin layer chromatography plates – these are normally sketched into lab notebooks, but a picture provides an interpretation-free presentation of the data that is more nuanced.^16^ Fig. 2 illustrates the use of photographic images in the recording of experiments conducted for the OSM project.

Some other groups use LabTrove templates to capture the details of procedures, rather than using other tools and adding the outcome to an associated notebook entry. Those researchers who do exploit templates find them convenient and easy to use; however, templates are not widely employed and experience with them has been mixed. Cameron Neylon uses templates extensively for his bioscience experiments^4^ but other groups are reluctant, as noted in the Metadata and templates section.

Data management is commonly a function of scale: data describing experimental conditions is usually incorporated in a notebook entry; the handling of raw and derived datasets depends on the volume of data involved. Datasets for many of the experiments conducted by the nanodroplets group are too large (sometimes many gigabytes) to be held on the Trove: the researchers upload only processed data, but link the relevant entry to the server location that holds the raw data. The Ultrafast X-ray project uses a similar strategy for very large files.

The flexibility of the ELN allows groups to make choices about the organisation of connected activities. The Todd group takes advantage of metadata keys to group related experiments together, particularly *post-hoc* as a classification tool. As an alternative approach, the biomaterials team uses separate notebooks to contain sub-projects that are facets of the overall project. Experiences accumulated from several laboratories highlight the need for training in best practices, particularly the most appropriate and efficient ways to use e-notebooks and metadata. Students regard LabTrove as intuitive to use, but accept that training is helpful when setting up standards for record keeping. Flexibility can sometimes be a mixed blessing, given the broad range of ways that researchers use the ELN, some of which are less effective than they could be.

## 3 Discussion of attitudes

The attitudes of ELN users in general and particularly their stance towards capturing metadata are almost impossible to separate from the viewpoints of LabTrove users, although the experiences described in this article relate primarily to that ELN. Willoughby *et al.* discuss in greater depth the opinions of users about ELNs and specifically their attitudes to capturing metadata.^13^

Paper continues to be attractive for capturing and preserving records of scientific activities, although ELNs are progressively replacing the traditional hardback books.‡
‡The authors of the review^1^ have recently become aware of the book entitled “Notebooks, English Virtuosi, and Early Modern Science”, by Richard Yeo, which shows how scientific note taking started and developed. It places our current discussions in the context of the development of research. [The University of Chicago Press, 2014, ISBN-10 0-226-10656-X].
^1^ Some researchers in Brynn Hibbert's group still find the immediacy of a paper notebook attractive, but this is by no means a general view. The majority of users refer only to the advantages of the ELN, particularly its central repository, which, for the biomaterials team, meant retrieval of useful data was fast and easy without having to file and go through lots of paper copies.

The issue of preserving the individuality of records is sometimes raised in comparisons between paper and electronic notebooks. LabTrove maintains a revision history for any modifications to an entry, with digital timestamps: users can examine that trace, which constitutes part of the provenance chain. In comparison, one researcher pointed out that paper records could be modified or doctored, even after the record has been signed and dated. Making such alterations without “showing the working” and thus demonstrating full provenance would raise issues reminiscent of the so-called “ClimateGate” affair.^24^

The overarching influence on the successful deployment of LabTrove, and probably ELNs in general, is the attitude of users to what content they are prepared to contribute to the ELN. Given the more private nature of a paper notebook, it is difficult to compare the extent to which users are willing to disclose information of a more thoughtful nature.

The Artemis project at the Central Laser Facility encountered some initial resistance from collaborators. Nevertheless, after three experiments, *the team had fully engaged in the process meaning almost all data and discussion was done via the* [project notebook], *thereby providing the useful narrative that is essential when looking back over data after a long time*. To begin with, the collaborators could not see the advantages, but now appreciate the benefits of being aware of developments as they occur and the ability to contribute fresh thoughts and perspectives.

There is evidence that experience with using a researcher-centric ELN can mitigate and even remove inhibitions. For example, the attitudes that the X-ray group reports are notably positive: the overall record transferred between different generations of researchers is much better, so information much less likely to be lost, and researchers continue to collaborate even when they are away.

With regard to access, the usual approach is to make the Trove private to the research group, although Mat Todd's projects involving open science provide the notable exceptions.^16^ However, inhibitions still exist for researchers working in an open domain, notably a desire to communicate by e-mail, rather than using an open form of communication.^14^ Another factor that may make people hesitant to use an open ELN is the worry associated with making a mistake that is visible to all, although the importance of this to a scientist may depend on the stage they have reached in their career.

Other inhibitors to using the ELN by students appear to be related to the lack of familiarity with ELN systems and the perceived complexity compared to using a paper notebook. Although neither specific to LabTrove, nor a universal opinion, using a paper notebook is perceived to be faster and more convenient, particularly for access and sharing. Moreover, digital media offer other advantages in terms of storage space, auditability, and significantly lower vulnerability to damage. It is clear that the benefits of the ELN are not generally understood, but many do appreciate the value of being able to link directly to their data. It is important to stress in training that an electronic notebook is still a notebook and entries are not judged by the quality of the write-up.

## 4 Devices

As noted in the preceding section, the immediacy of a paper notebook remains attractive in a laboratory context: it can be inconvenient to move to a desktop computer, obtain access, and then type the necessary information but this would not seem to have presented a significant barrier to industrial users. Tablet computers offer a form of compromise that the Smart Tea system explored, using a tablet PC that could be carried around in the laboratory.^25^

The affordances of the book we wanted to translate to the digital were: ease of access for flipping between previous and concurrent experiments; simple data entry for measures, free form sketches and annotations; portability in the lab, and secure data storage.^26^

In a wet lab chemical or pharmaceutical environment it is preferable not to move the research record between lab and clean area environments for fear of contamination; clearly this is straightforward with a digital record.

In the preliminary stages of adopting LabTrove, Brynn Hibbert experimented with early notebook computers that had some stylus capability (HP EliteBook) and with Blackberry PDAs, yet still found that paper notebooks were more popular. This reluctance could be attributed partly to poor connectivity. Tablet and mobile technologies have moved on considerably since the early trials with capturing observations and data in the laboratory.

A fundamental characteristic of ELNs is the ability to capture and store data in a machine-readable form, thus offering a clear advantage over paper notebooks. LabTrove has the capability for attaching data files of a wide variety of formats to notebook entries. Each data file added to the Trove has its own URL; it is also possible to link to data files stored externally.

Inspection of the open notebook for the Open Source Malaria project shows that the consortium makes extensive use of the attached-data capability:^16–18^

*“Lastly are posted data (NMR, IR, HRMS,* etc*.), The processed form of the data, suitable for human interpretation, is usually posted in PDF format, while the raw data are posted in a variety of formats, and are included so that they may be accessed and processed by anyone.”*

Some groups have implemented fully automated transfer, using purpose-built programs that extract data from a device, or process the results of a computation, and employ LabTrove API calls to create notebook entries. We discuss such approaches in more detail in the API-based applications section. As well as extending the capability of the ELN, such data transfer is also in some respects characteristic of a Laboratory Information Management System (LIMS).

Cameras and mobile phones are used increasingly to capture visual and audio records of experiment artefacts. The data files can be uploaded to a computer and added as a data item to a notebook entry. As noted previously, the size of data files might restrict what is actually uploaded; it might be appropriate in some cases to create a compressed (zip) file. Digital pen files and voice recording devices could also be used and with the expanding Internet of Things,^27,28^ the nature of inputs is likely to become even more diverse.

During the early development of LabTrove, one dyslexic student at the University of Southampton pioneered the use of visual recording in his notebook. Such usage is now routine, notably by the Todd group, who use camera phones and Wi-Fi-enabled SD cards for capturing experiment artefacts visually, as described in Section 2.3.

Tablet devices offer the opportunity to combine several forms of recording, including textual notes captured at source, and to interface directly with the ELN. Willoughby *et al.* assess the evolution of ELN tablet interfaces, covering also their provisions for capturing experiment plans and observations, which can subsequently be exported to the ELN.^29^

## 5 Specific application areas

In this section, we give an overview of seven research areas in which LabTrove has been applied for the benefit of the project to provide recording and collaboration facilities. The Trove might or might not also contain notebooks for individual researchers.

### 5.1. Ultrafast X-ray project

The Ultrafast X-ray project involves researchers from Physics, the *Optoelectronics Research Centre* (ORC), Chemistry, and Biology at the University of Southampton. The team develops laser-based systems to generate short-pulsed soft X-ray sources for spectroscopy and imaging.

The Trove records narrative about plans, equipment, experiments, and outcomes. Data are captured as images and spreadsheets as well as in its raw form, some being added automatically, not only during the experiments but also as the result of MATLAB computations. Literature sources and draft papers are also held in the Trove, for collaboration purposes. Researchers are strongly encouraged to add material daily and certainly as a summary prior to each weekly group meeting, especially as those meetings are run from the Trove, by going through the entries added since the previous meeting.

This project clearly uses the Trove as a record and collaboration system, which the Principal Investigators believe has changed the way the project is run, in such a way that the team have come to rely on LabTrove.

### 5.2. Crystallography

**Fig. 3:**
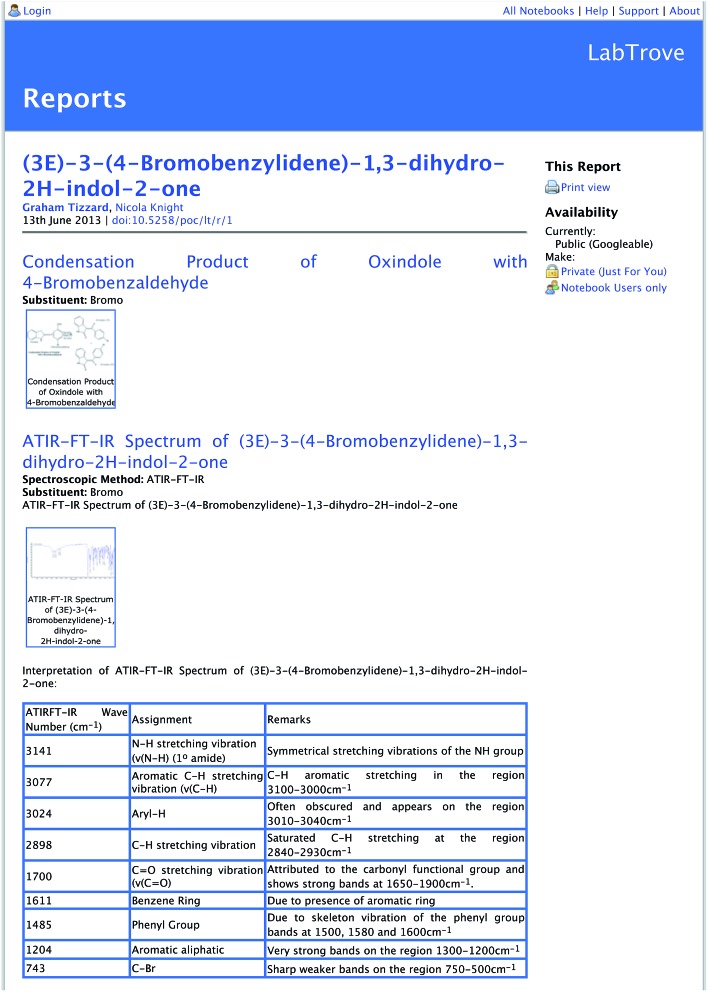
The top part of the landing page for the DataCite DOI (http://data.datacite.org/10.5258/POC/LT/R/1) for the synthesis and characterisation of substituted indoles. This landing page is a LabTrove ELN entry and has links to further more fine grained data: Graham Tizzard; Nicola Knight; (2013): (3*E*)-3-(4-Bromobenzylidene)-1,3-dihydro-2*H*-indol-2-one; LabTrove – University of Southampton.

We outlined the collaborative nature of a crystallographer's work when discussing how various groups deploy LabTrove. The UK National Crystallography Service (NCS) uses LabTrove to consolidate the results of synthesis and characterisation work, such as spectroscopy, into a single resource that supports the publishing process, as illustrated by the example in Fig. 3.

Collaboration of this nature, which involves service work, presents several challenges:

• understanding the provenance of data and materials, particularly the history of samples and the methods employed in their synthesis;

• collating the results of complementary techniques when trying to understand a crystal structure or to discuss aspects with the originator of the sample;

• maintaining an open dialogue with collaborators about the work itself and the suitability of the results for publication;

• creating cohesive publications for which individuals working in isolation write sections.

The NCS recognised that the ELN had the potential to address some of these issues, so undertook an active investigation. The outcomes to date indicate that the ELN has shown its worth, especially as it has changed the mind-set of researchers, who now capture vital information where they would not have done so in the past.

A key requirement of the publishing process is to generate a persistent component of the scientific record.^30^ The University of Southampton has a contract with DataCite, which is an international body set up to enable the use of persistent Digital Object Identifiers (DOIs) to support the preservation, citation, discovery, and reuse of data^31^ in a similar manner to DOIs for papers. The NCS has developed a LabTrove plugin that generates a dataset in report form, which can be used to mint and register a DataCite DOI for the dataset. Fig. 3 shows the top part of the ELN report that forms the landing page for the DataCite DOI;^32^ the corresponding eCrystals repository record, the crystal structure determination for the compound reported, and the description of the synthesis are available separately.^33,34^

### 5.3. Open Source Malaria (OSM)

As demonstrated by the UK Crystallography Service, ELNs can be powerful enablers of multi-disciplinary and cross-institutional projects. The need to be explicit about context for audiences less familiar with the subject matter in an inter-disciplinary research project provides a powerful push towards the use of an ELN.

The Todd group leads an open source cross-disciplinary medicinal chemistry project aiming to find new medicines for malaria, which uses an entirely public Trove^14^ in addition to a number of other tools. The OSM project involves a wide range of data types and formats: some entries relate to synthetic organic chemistry; other notebooks focus on biological evaluations of compounds or molecular modelling. The Trove also contains summary notebooks, collated for easier submission for publication or for sharing with potential collaborators.

The OSM Trove comprises a range of notebooks that are all accessible from the main dashboard. In some cases, for example where a notebook is used for a student assessment, the notebook has a single author. In other cases multiple authors share a notebook, such as for collaborative sub-projects. One member of the team, Alice Williamson, has written a *How-To* guide for creating an OSM notebook entry, which provides a comprehensive introduction that ranges from getting started through attaching data and image files to publishing from the notebook.^35^ This was an important tool for those outside the project, unused to LabTrove, to help them contribute directly through the writing up of their own experiments, which has happened in a number of cases.^36,37^

### 5.4. Australia National Data Service

The Australia National Data Service^37^ funded UNSW to create a secure method for storing raw data from instruments in the Analytical Centre (for example, spectrometers, microscopes) and for creating metadata to comply with the Government's requirements to track the results of scientific research. The group developed the Analytical Centre Data Management System (ACData) as a web-based tool to enable researchers to capture and curate research data from selected analytical instruments at UNSW.^38^ The Hibbert group was funded to extend this curation to push data to the ELN for its users. Notebook entries comprise attached data files as well as the required metadata in the form of key-value pairs, which comprise an identifier label and a specific value for that identifier.

### 5.5. Selectivity

As researchers in various institutions become familiar with the capabilities of LabTrove, they are developing use cases that involve a higher degree of selectivity at both the capture and the archival stages. From the early stages of its development, the system adopted a *one-item one-entry* (originally *one-item one-post*) approach, owing to the need (perceived at the time) uniquely to identify each element of the research record, such as data, samples, and protocols.^4^ However, the developers adopted a compromise approach for repeated procedures, particularly when conducted in parallel, opting to keep sets of procedures together in one container entry.

Several groups have requirements that involve selecting a set of entries from a notebook to generate a collection that can be passed to a data repository or to another application. The UK Crystallography Service tool that creates datasets for DataCite is one example; the UNSW ACData tool is another. The export of selected entries to produce a single file for subsequent archival to a data repository seems almost certain to become a generic use case. Such exports will be particularly relevant when the request is to provide a provenance chain, for example, to comply with regulatory purposes, as a constituent part of the publishing process, or for review purposes. The ELN preserves the provenance of procedures, data, and other artefacts by maintaining links between the objects in notebooks and by enabling users to capture the appropriate metadata. Links and metadata can be used individually or in combination to enable selective retrieval of entries for inspection and potentially archiving.

### 5.6. Teaching and learning

Brynn Hibbert and Rosanne Quinnell collaborated in a project to investigate the use of LabTrove in undergraduate laboratory teaching in chemistry. This project ran from 2010 to 2012 and was funded by the Australian Learning and Teaching Council (now the Office of Learning and Teaching (OLT)). A noteworthy feature of this project was its creation of a global chemistry “collaboratory” for undergraduate science students at UNSW, Curtin University (Western Australia), the University of Sydney, the University of Southampton, and Chiang Mai University (Thailand). Further information about this international collaboration is available from the project website.^39–41^

An exciting outcome was to show how an experiment that is relevant to all countries, for example measuring mercury residues in fish, could be set up across different institutions, allowing the students to drive the learning experience. The asynchronous nature of the ELN supported incremental additions to the results of an experiment. The ELN was never used by an active undergraduate cohort, and organising several institutions to test out experiments was very difficult. However a series of two-centre collaborations were established with students and staff developing and testing experiments. With appropriate planning before courses start, the problems of time zones, different curricula, and different levels of available equipment, can be overcome. Subsequently a large undergraduate cohort in the University of Southampton's extended year 3 practical course has used LabTrove and a detailed analysis of this is being prepared for publication.^42^

Some students currently using LabTrove have experience of working in industry as well as academia, and have used both paper and electronic laboratory notebooks. While they all agree that using an ELN enables them to maintain good records, one student expressed the view that “*academia is lagging far behind and not teaching the skills required in industry*.” However while we have been able to discuss the impact of the ELN on the students' university research, the available sample of students going on to industry is too small at this stage to be able to comment sensibly on the wider impact of this preparation. As our user base increases we will monitor the effectiveness of academic training in using an ELN as preparation for record keeping in industry.

### 5.7. API-based applications

For several of the application areas described, teams have automated the transfer of data to the project notebook. In some cases, an instrument generated the data transferred; in other cases the data was the result of MATLAB computations. The Ultrafast X-ray group has written an “autoblogger” that uses API calls to upload files to their Trove, for example the computer controlling the X-ray cameras writes the raw data files to a server and a directory watcher “notices” when new data files have been added and then creates a new notebook entry in the camera notebook and adds the data file (or a link to the file on the server) and converts the data to a jpg image which is attached to the notebook entry allowing the group to review the data very quickly.

Scientists at the STFC Rutherford Appleton Laboratory (RAL) have deployed LabTrove for tracking experiments conducted at the large scale STFC ISIS neutron source facility and the subsequent processing of the data collected. Their work is in response to increasing interest in improving the connectivity of the data collection process with the downstream data processing pipelines.

**Fig. 4:**
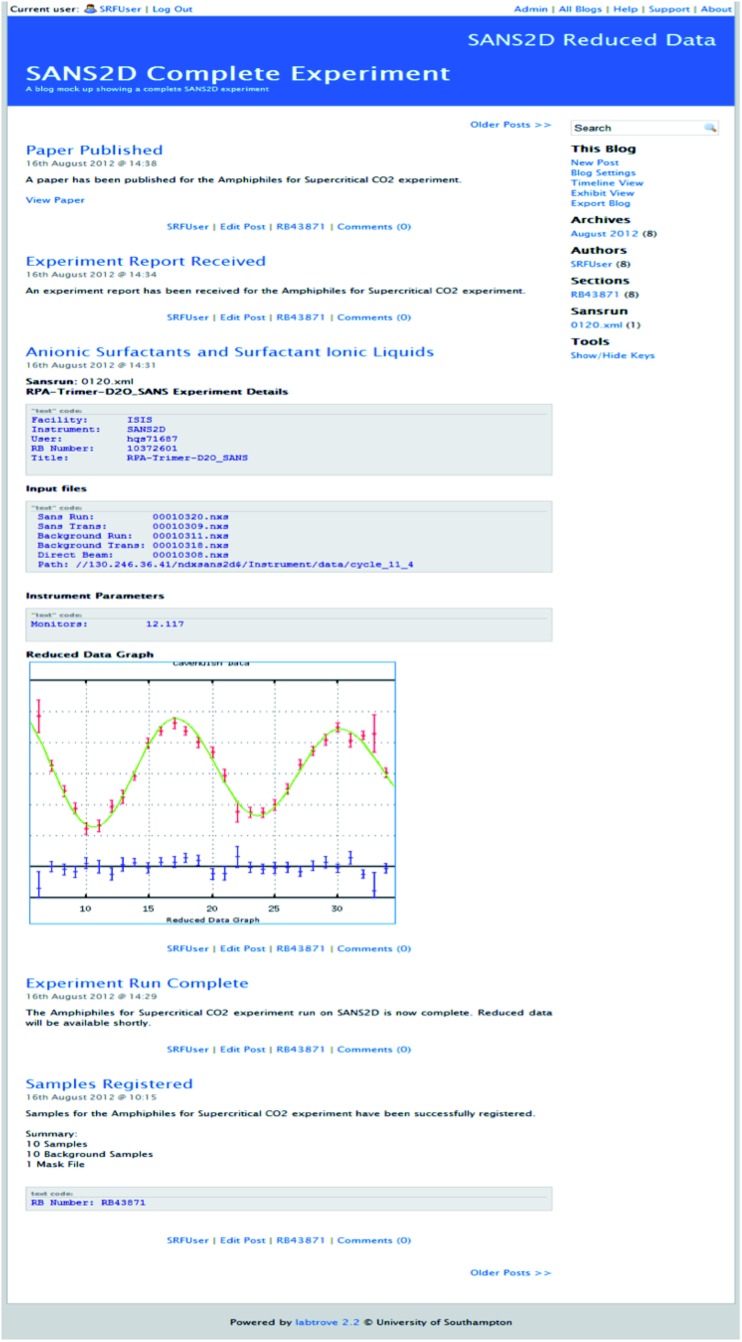
Notebook entries for time-of-flight experiments with the Small-Angle Neutron Scattering instrument (SANS2D) at the ISIS UK neutron facility.^44^ These entries illustrate integration of the LabTrove ELN with ISIS experiment control and the STFC ICAT data infrastructure at the Rutherford Appleton Laboratory (RAL).^45,46^

The ISIS production data management infrastructure relies on a metadata repository that stores the metadata about an experiment, covering the facility lifecycle prior to publication. Fig. 4 is an example of LabTrove usage, showing a series of notebook entries that were created *via* API calls, thus capturing experiment lifecycle events such as: sample registration; end of experiment run; raw data capture; reduced data available; experiment report sent; and paper published. The ELN is also used to record automatically the data reduction workflow for a live experiment and the data provenance for the reduction workflow.^43^

The benefits that the STFC scientists obtained by deploying the ELN include the immediate availability from a single location of automatically aggregated data and other experiment-related information.

Within the ELN development team, we have experimented with an API-based tool for recording a multi-step experiment in a single container entry. With the web interface, notebook entries for repeated procedures can be kept together in one container entry. To enable the API-based tool to capture the results of an individual step in a multi-step procedure, we developed an extension to the API library for appending to an existing notebook entry.

## 6 Metadata and templates

In our complementary publication, we present the results of a survey of metadata usage in LabTrove notebooks together with an analysis of user perceptions of and expectations for metadata in digital notebooks, gathered through user interviews and user testing activities of different groups.^13^

The metadata survey and the user experience feedback demonstrate that the biggest inhibitor to the capture of useful metadata is the *blank canvas effect*. Although users are willing to add metadata, they do not know where to start. Many laboratory researchers will not know what “metadata” means, and so will not appreciate why they should capture it.

Some of the projects described in the Specific application areas section have requirements for metadata capture and for the vocabulary to be used. One objective of the UNSW ACData system was to create metadata to comply with the Australian Government's desire to track the results of scientific research. However, the system did not mandate or prescribe particular metadata schema, owing to a lack of certainty about which schema best applied, as the terminology varied from discipline to discipline. Accordingly, the metadata captured with ACData is that automatically generated by the instruments during the data generation process, but researchers also have the ability to add any additional metadata they feel is necessary as key-value pairs.

In the STFC application the experiment identifier is stored as a Section value, thus providing access to the experiment provenance. The ISIS specific proposal number, given at the point of proposal approval, is used to link up the events. Additional metadata, as simple key-value pairs, are stored alongside the entries to indicate the type of event an entry relates to.

The Ultrafast X-ray group makes significant use of Sections and, as users become familiar with LabTrove, an increasing use of key-value pairs. The main purpose for the key-value metadata is to aid search and linking. The Principal Investigator believes that the team could make more use of such provisions, for example to mark up a narrative history.

The Mueller group makes extensive use of metadata key-value pairs to characterise notebook entries, particularly to enable grouping of entries by sample information. The group uses the section field to indicate the nature of each entry, for example, settings, experimental conditions, samples, and programs.

Brynn Hibbert has also tried to arrange training for researchers in how to set up metadata in their notebooks, but has experienced difficulties owing to the lack of a common approach that would suit all projects.

The team at the University of Southampton have developed a technique for preloading a notebook with key-value pairs that project leaders can incorporate into templates. This technique enables projects that require a more controlled metadata vocabulary to specify the keys that they expect researchers to provide and the range of values that they can use.^13^

None of the projects that we appraise in this article make extensive use of LabTrove templates, although Brynn Hibbert acknowledges that they offer useful ways of ordering information that is repeated and for documenting items that are used frequently, such as chemicals and electrodes. The limited use of templates can to some extent be attributed to the ease of creating LabTrove entries in a standardised form. However, as researchers have become more accustomed to using the ELN and to searching and reusing the information therein, there is increasing recognition of the merits of templates. We expect the deployment of templates to expand in the future.

**Fig. 5:**
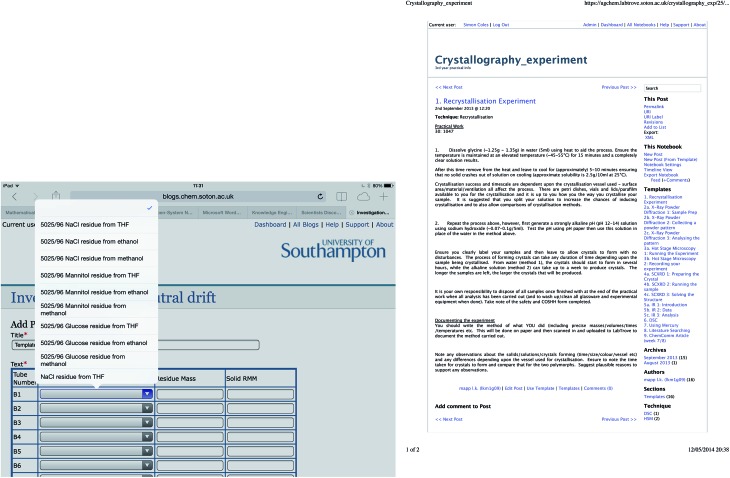
An example of a LabTrove formal template to facilitate linking between entries with automatically generated drop down menus (see PLoS paper for more details and a “template” set of instructions from the undergraduate notebook describing an step in an undergraduate practical).^42^

The very recent undergraduate trial in Southampton has made extensive use of templates,^47^ as did the biological chemistry cases that inspired much of the design of the ELN and which are discussed in the PLoS paper.^4^ Fig. 5 shows an example template from the undergraduate notebook.

## 7 Future

In this section we review the future requirements that users of the ELN have identified and consider the implications for both LabTrove and ELNs in general.

### 7.1. Metadata capture

*Improve metadata capture to obtain better metadata input*. Appreciation of the importance of metadata is undoubtedly increasing, although some researchers remain unsure about the best approach to organising metadata within their ELN. Our complementary publication includes a comprehensive discussion of approaches to addressing both the inhibitors to better metadata usage and to improving knowledge about the nature and importance of metadata.^13^

Two users responded to the request to suggest enhancements by proposing a role for librarians in improving metadata usage, which would seem to resonate with the views of Losoff, who discusses the potential value of librarians in data curation.^48^ She observes:

*“Scientific progress increasingly relies on searchable and intelligent integration of data sets, mined in conjunction with journals and other resources.”*

There is growing interest in the involvement of librarians in training researchers in data and information management and the increased role of information and data specialists in helping train the next generation of scientific researchers can only be to the good.

### 7.2. Capture of structured data

*Improve the means for entering data such as reaction schemes and tables of values*. Users currently overcome the lack of support for entering structured data by attaching representations of that data to notebook entries. The approach taken by Mat Todd's group is illustrative and typical: reaction schemes are drawn using ChemDraw^22^ then saved and embedded as PNG files; tables containing names, quantities and other important values of the chemicals used in a reaction are constructed in Excel and added as PNG files. Such conversions reduce the value of the chemical and quantitative information contained in the schemes and tables.

Current versions of the ELN require a researcher to create a notebook entry before attaching any data files to that entry. Penn State in particular would like to see this process enhanced so that data can be uploaded and attached during the initial creation of the notebook entry and for images of TIFF, JPG, and PNG files to be embedded automatically, to make them visible when reading the entry. Several users have indicated that they would like a drag and drop feature for attaching data files to a notebook entry.

It is evident from the development of digital notebooks in general that users expect more capability than the recording of text and the storage of uploaded files. The capture of data structured according to the requirements of the application area is increasingly important.

### 7.3. Openness and open source

The open source and web based nature of LabTrove was significant in allowing the adoption of the software by a geographically disperse set of research groups without the need for extensive licence negotiations, which was attractive to individual groups. This is true of any of the open source solutions, but it must not be thought that running the service is without cost even for “free software”. The software always needs to be hosted somewhere and there are always running costs even if no licence costs. This is though a strong driving force as noted by Elliot.^49^

The lack of need for intellectual property protection and limited funding are stimulating an upsurge in the use of open source software and low-cost tools like Evernote.

Atrium Research makes available a list of suppliers of ELN and ELN-like products, which includes a section devoted to open source ELNs.^50^ The list is also instructive with regard to the range of domains in which ELNs are now employed.

The browser interface maintains researcher centricity by allowing users to deploy their local software. For example, when downloading a data file from the ELN, the file type will enable the user automatically to launch the relevant software that is associated with that type of file. While a data file of an unknown type could cause a problem, we note that the ELN system itself does not try to offer all functionality to all users; it simply enables them to employ their usual software. Such considerations make an open source solution attractive. While we would comment that open source does not necessarily mean there are issues with IP protection, it is sensible to highlight a potential issue for wider scale adoption. Software generated for small-scale use does not always scale up for an enterprise, for example, from use by a research group to adoption by the whole university. Clearly much open source software can be used at small-scale and as an enterprise solution but this does impose a new set of requirements, as identified in the evaluation report produced by the University of Wisconsin–Madison.^51^

Discussions about linking in to the enterprise authentication system to provide single sign-on always take time. When an enterprise system has been adopted, allowing access to colleagues outside the enterprise can be more problematic from an organisational administrative perspective (not the software), as shown by the demise of many grid-type services. The move to cloud solutions does not necessarily help, as this is not a matter requiring a technical solution. The issue of enterprise readiness was one of the important aspects raised by the Wisconsin report. On the basis of the experiences reported here we would highlight even more the interplay between the requirements of global collaboration and internal enterprise protection.

## 8 Conclusions

Progress towards electronic research notebooks becoming the preferred, if not mandated, medium for scientific record keeping is inexorable, although still uneven.^1^ Key drivers for the move to electronic recording in the academic environment are the research data management requirements of funding bodies and a justifiably increased focus on accountability. Even drivers as powerful as these can nevertheless meet with resistance. Most academic institutions are moving towards the use of ELNs in research, driven by regulatory requirements, particularly the mandates to store data in open repositories. Although there is still inertia, even within schools that have groups already using ELNs, it seems likely that improvements in technology will lead eventually to ubiquitous adoption.

We have shown that the open source LabTrove software is a versatile electronic notebook system that works well for individual and especially collaborative projects, encouraging better data management practice. It provides an auditable provenance trail for data in context, and can be moulded in a variety of ways to suite the individual users. We highlight two aspects of the software. Firstly the design focus is to provide a set of easy to use functions that meet the basic needs of any researchers, concentrating on the commonalities of the researchers rather than the research topics. The researcher takes centre stage, and they are given the ability to arrange the notebooks to suit their own needs and organisational views. This is also reflected on a larger stage with the adoption of an open source approach. The software costs to individual researchers are minimised, The ELN can be readily used in widely dispersed collaborations without complex licence negotiations, and the community can add new features to the software.

Our collation of differing practices in the usage of LabTrove has led us to reflect upon the architecture required for a generic digital notebook appropriate for researchers in a wide range of disciplines. We envisage such a digital research notebook (DRN) having a core set of capabilities, including but by no means confined to: data management; access control; and provenance inspection. The generic DRN would be capable of being specialised for specific disciplines, for example chemists would require facilities for structure drawing and search. Any DRN will be more than a content management system and so must cater for the social as well as the technical aspects of research projects. We have emphasised the journal characteristics of research notebooks and it is important not to lose sight of such provisions when implementing a DRN.

We do recognise the complex socio-technical problems in encouraging the use of metadata, which is fundamental to the really high value use and re-use of information. Capture of the best metadata that would facilitate recall, discovery and reuse is still a difficult task. The nature of recording does require significant after-the-event entries, although the ability to interact with the ELN *via* the Web using mobile devices does enable “at the bench” recording and facilitating this is on the roadmap for moving LabTrove forward.

The research groups involved in the work reported here had different reasons for adopting a digital notebook and for choosing LabTrove in particular. One major theme was about sharing information: within a research group between student and supervisor; as part of a group (interdisciplinary) project; to provide access to the raw data in context on publication; or more widely as part of a more open approach to research.

## References

[cit1] Bird C. L., Willoughby C., Frey J. G. (2013). Chem. Soc. Rev..

[cit2] Elliott M. H., New Debates over Intellectual Property Protection and ELN, http://www.scientificcomputing.com/articles/2011/01/new-debates-over-intellectual-property-protection-and-eln?terms=electronic%20laboratory%20notebook#.UnDseiR0NGt, accessed March 2014.

[cit3] Hibbert D. B. (2014). J. Proc. R. Soc. N. S. W..

[cit4] Milsted A. J., Hale J. R., Frey J. G., Neylon C. (2013). PLoS One.

[cit5] Ultrafast nanoscale X-Ray scattering, https://www.southampton.ac.uk/chemistry/research/projects/ultrafast_nanscale_xray_scattering.page, accessed April 2014.

[cit6] Neylon (Director) C., Investigations into neutral drift, https://blogs.chem.soton.ac.uk/neutral_drift, accessed October 2014.

[cit7] Marazzi L., Chemistry project (year 3), https://blogs.chem.soton.ac.uk/luke_marazzi, accessed October 2014.

[cit8] ModTrove, https://github.com/miike/modtrove, accessed March 2014.

[cit9] LabTrove. http://www.labtrove.org/, accessed October 2014.

[cit10] Open Notebook Science. UsefulChem, http://usefulchem.wikispaces.com/, accessed October 2014.

[cit11] Bradley J. C. (2007). Nature Precedings.

[cit12] Frey J. G., in Abstracts of Papers, 244th ACS National Meeting & Exposition, American Chemical Society, Philadelphia, PA, 2012.

[cit13] Willoughby C. Bird C. L. Frey J. G. Coles S. J., J. Chem. Inf. Model., 2014, , submitted . 10.1021/ci500469f 25405258

[cit14] Robertson M. N., Ylioja P. M., Williamson A. E., Woelfle M., Robins M., Badiola K. A., Willis P., Olliaro P., Wells T. N. C., Todd M. H. (2014). Parasitology.

[cit15] Open Source Malaria. http://openwetware.org/wiki/Open_Source_Drug_Discovery_-_Malaria, accessed October 2014.

[cit16] Pictet-Spengler route to Praziquantel, http://www.ourexperiment.org/racemic_pzq, accessed March 2014.

[cit17] Woelfle M., Olliaro P., Todd M. H. (2011). Nat. Chem..

[cit18] Woelfle M., Seerden J.-P., de Gooijer J., Pouwer K., Olliaro P., Todd M. H. (2011). PLoS Neglected Trop. Dis..

[cit19] OSM – Open Source Malaria, http://opensourcemalaria.org, accessed March 2014.

[cit20] The Mueller group at Penn State, http://research.chem.psu.edu/ktmgroup/, accessed March 2014.

[cit21] Badiola K. A. Quan D. H. Triccas J. A. Todd M. H., PLoS One, 201410.1371/journal.pone.0111782 , , in press . 10.1371/journal.pone.0111782 PMC4262224 25493550

[cit22] ChemDraw, http://www.cambridgesoft.com/Ensemble_for_Chemistry/ChemDraw/, accessed March 2014.

[cit23] http://www.ourexperiment.org/racemic_pzq/3955/Partial_PS_reaction_to_give_the_PZQ_enediamide_intermediate_KAB131.html, accessed March 2014.

[cit24] Hulme M. and Ravetz J., http://news.bbc.co.uk/2/hi/science/nature/8388485.stm, accessed March 2014.

[cit25] Hughes G., Mills H., De Roure D., Frey J. G., Moreau L., schraefel m. c., Smith G., Zaluska E. (2004). Org. Biomol. Chem..

[cit26] schraefel m. c., Hughes G., Mills H., Smith G., Payne T. and Frey J., Breaking the book: translating the chemistry lab book into a pervasive computing lab environment, in Proceedings of ACM CHI 2004 Conference on Human Factors in Computing Systems, ed. Dykstra-Erickson, Elizabeth and Tscheligi, Manfred, Vienna, Austria, April 24-29, 2004, pp. 25–32.http://dl.acm.org/citation.cfm?doid=985692.985696, accessed October 2014.

[cit27] The Internet of Things Council. http://www.theinternetofthings.eu/, accessed October 2014.

[cit28] Ferber S., How the Internet of Things Changes Everything, http://blogs.hbr.org/2013/05/how-the-internet-of-things-cha/, accessed October 2014.

[cit29] C. Willoughby, J. G. Frey and C. L. Bird, The evolution of ELN mobile & tablet interfaces, in preparation October 2014.

[cit30] Digital Curation Centre (DCC), Overview of funders' data policies, http://www.dcc.ac.uk/resources/policy-and-legal/overview-funders-data-policies, accessed March 2014.

[cit31] DataCite. http://www.datacite.org/, accessed March 2014.

[cit32] LabTrove Report, http://poc.labtrove.soton.ac.uk/report/1, accessed March 2014.

[cit33] eCrystals, Bromo Oxindole, http://ecrystals.chem.soton.ac.uk/1323/, accessed March 2014.

[cit34] Tizzard G. J., Coles S. J., Edwards M., Onyeabo R. O., Allen M., Spencer J. (2013). Chem. Cent. J..

[cit35] Williamson A. E., http://malaria.ourexperiment.org/osm_logos_and_templ/7773, accessed March 2014.

[cit36] Biological Evaluation of Compounds, http://malaria.ourexperiment.org/biological_data/7840, accessed March 2014.

[cit37] Australian National Data Service. http://ands.org.au/, accessed March 2014.

[cit38] Cox S., Leslie G., Hibbert D. B., Moran G. and Hawkins N., eResearch Australasia, Sydney, 2012, http://conference.eresearch.edu.au/eres2012/, accessed March 2014.

[cit39] Quinnell R., Hibbert D. B. and Milsted A., Same places, different spaces. Proceedings ascilite, Auckland, 2009, http://www.ascilite.org.au/conferences/auckland09/procs/quinnell.pdf, accessed March 2014.

[cit40] Hibbert D. B., Frey J., Quinnell R., Mocerino M., Todd M., Niamsup P., Plummer A. and Milsted A., Teaching instrumental science globally using a collaborative electronic laboratory notebook, Proceedings of the 16th UniServe Science Annual Conference, The University of Sydney, Sydney, Australia. http://www.itl.usyd.edu.au/aboutus/2010%20conf%20proceedings%20final.pdf, accessed October 2014. Poster available from http://www.academia.edu/1152964/Extending_the_science_curriculum_teaching_instrumental_science_at_a_distance_in_a_global_laboratory_using_a_collaborative_Electronic_Laboratory_Notebook.

[cit41] Quinnell R. and Hibbert D. B., Introducing an Electronic Laboratory Notebook to PhD Students Undertaking Chemistry Research at A Research Intensive University. The International Conference on Society and Information Technologies, Orlando, Florida, USA, 2010. http://www.iiis.org/CDs2010/CD2010IMC/ICETI_2010/PapersPdf/EB108QJ.pdf, accessed March 2014.

[cit42] Coles S. J., Private Communication.

[cit43] ISIS reduction workflow, SRF: Capturing data provenance trails for science, http://www.stfc.ac.uk/e-Science/projects/medium-term/38418.aspx, accessed April 2014.

[cit44] ISIS. Science & Technology Facilities Council. http://www.isis.stfc.ac.uk/, accessed October 2014.

[cit45] ICAT Project. http://icatproject.org/, accessed October 2014.

[cit46] Matthews B. and Bicarregui J.. Towards an open data infrastructure for photon and neutron facilities, http://pan-data.eu/sites/pan-data.eu/files/DigitalResearch2012-PanData.pdf, accessed October 2014.

[cit47] LabTrove ELN for Chemistry undergraduates (restricted access), https://ugchem.labtrove.soton.ac.uk/, accessed March 2014. Access to these notebooks is currently restricted to the Southampton Domain please contact the authors to arrange access.

[cit48] Losoff B., Issues in Science and Technology Librarianship, 2009 10.5062/F4HH6H0D.

[cit49] Elliott M. H., http://www.scientificcomputing.com/articles/2014/03/state-eln-current-perceptions-and-new-paths, accessed March 2014.

[cit50] Atrium Research – Electronic Laboratory Notebooks, http://www.atriumresearch.com/html/eln.htm, accessed April 2014.

[cit51] Electronic Lab Notebook Pilot at the University of Wisconsin-Madison, http://academictech.doit.wisc.edu/files/ELN_pilot_report_UWMadison.pdf, accessed March 2014.

