# Supplementary material for: Experiences with a researcher-centric ELN
Source: Chem Sci. 2014 Oct 20;6(3):1614–29. doi: 10.1039/c4sc02128b (PMC5639792; doi:10.1039/c4sc02128b)
Supplement: Supplementary file 1 [file SC-006-C4SC02128B-s001.pdf]

Electronic Supplementary Material (ESI) for Chemical Science.
This journal is © The Royal Society of Chemistry 2014

Experiences with a researcher-centric ELN

List of abbreviations used in the paper

ACData	Analytical Centre Data Management System
CLF	Central Laser Facility
DRN	Digital Research Notebook
ELN	Electronic Laboratory Notebook
HRMS	High Resolution Mass Spectrometry
ICAT	http://icatproject.org/
IR	Infra-red
ISIS	http://www.isis.stfc.ac.uk/
LIMS	Laboratory Information Management System
NCS	Nation Crystallography Service
NMR	Nuclear Magnetic Resonance
OLT	Office of Learning and Teaching
ORC	Optoelectronics Research Centre
OSM	Open Source Malaria
RAL	Rutherford Appleton Laboratory
SANS2D	Small Angle Neutron Scattering instrument
STFC	Science & Technology Facilities Council
UNSW	University of New South Wales
